# New genetic variants of *LATS1* detected in urinary bladder and colon cancer

**DOI:** 10.3389/fgene.2014.00425

**Published:** 2015-01-13

**Authors:** Mona K. Saadeldin, Heba Shawer, Ahmed Mostafa, Neemat M. Kassem, Asma Amleh, Rania Siam

**Affiliations:** ^1^Biotechnology Department, American University in CairoNew Cairo, Egypt; ^2^National Cancer Institute, Cairo UniversityNew Cairo, Egypt; ^3^Clinical Pathology Department, Cairo UniversityNew Cairo, Egypt; ^4^Biology Department, American University in CairoNew Cairo, Egypt; ^5^YJ-Science and Technology Research Center, American University in CairoNew Cairo, Egypt

**Keywords:** urinary bladder cancer, colon cancer, LATS1, genetic variants, tumor suppressor

## Abstract

*LATS1*, the large tumor suppressor 1 gene, encodes for a serine/threonine kinase protein and is implicated in cell cycle progression. *LATS1* is down-regulated in various human cancers, such as breast cancer, and astrocytoma. Point mutations in *LATS1* were reported in human sarcomas. Additionally, loss of heterozygosity of *LATS1* chromosomal region predisposes to breast, ovarian, and cervical tumors. In the current study, we investigated *LATS1* genetic variations including single nucleotide polymorphisms (SNPs), in 28 Egyptian patients with either urinary bladder or colon cancers. The *LATS1* gene was amplified and sequenced and the expression of *LATS1* at the RNA level was assessed in 12 urinary bladder cancer samples. We report, the identification of a total of 29 variants including previously identified SNPs within *LATS1* coding and non-coding sequences. A total of 18 variants were novel. Majority of the novel variants, 13, were mapped to intronic sequences and un-translated regions of the gene. Four of the five novel variants located in the coding region of the gene, represented missense mutations within the serine/threonine kinase catalytic domain. Interestingly, *LATS1* RNA steady state levels was lost in urinary bladder cancerous tissue harboring four specific SNPs (16045 + 41736 + 34614 + 56177) positioned in the 5′UTR, intron 6, and two silent mutations within exon 4 and exon 8, respectively. This study identifies novel single-base-sequence alterations in the *LATS1* gene. These newly identified variants could potentially be used as novel diagnostic or prognostic tools in cancer.

## Introduction

The large tumor suppressor gene (*LATS1*), encodes for a serine/threonine protein kinase. It was first identified in *Drosophila melanogaster* and later two mammalian homologs *LATS1* and *LATS2* were identified (Yabuta et al., 2000, 2007). LATS1 shares functional and structural similarities with LATS2. They are core components of the Hippo signaling pathway that promotes tissue growth and regulates cell proliferation. Mutations in the Hippo signaling pathway have been associated with tissue overgrowth and development of tumors. In mammals, the activation of the Hippo signaling inhibits cell proliferation through activation of the mammalian Ste20-like serine/threonine kinases 1 and 2 (MST1/2) that phosphorylates LATS1/2. Phosphorylated LATS1 in turn activates selected oncogenes such as yes-associated protein (YAP) or its paralog, the transcriptional co-activator with PDZ-binding motif (TAZ) (Hao et al., 2008). The phosphorylated form of YAP gets sequestered in the cytoplasm resulting in suppression of cell proliferation (Harvey and Tapon, 2007; Oka et al., 2008).

LATS1 regulate cell cycle by inhibiting the activity of CDC2 kinases during mitosis. The N-terminus of the phosphorylated LATS1 protein binds to CDC2 and reduces the activity of H1 histone kinase (Tao et al., 1999) leading to G2-M arrest (Xia et al., 2002). The phosphorylated LATS1 was reported to inhibit actin polymerization by binding to F-actin (Visser-Grieve et al., 2011). In agreement with LATS1 role as a tumor suppressor, its overexpression was observed to induce apoptosis by up-regulating Bcl-2-associated X (BAX) protein and caspase-3 pro-apoptotic proteins independent of P53 (Xia et al., 2002). The tumor suppressor LATS1 is down regulated in various soft tissue sarcomas (Hisaoka et al., 2002), and LATS1 down-regulation was shown to be a poor prognostic factor in glioma cases (Ji et al., 2012). Moreover, *Lats1* knockout mice induced the formation of ovarian tumors and soft tissue sarcomas (St John et al., 1999). Down-regulation of *LATS1* expression due to its promoter hyper-methylation was also observed in breast cancer, colorectal cancer and astrocytoma (Takahashi et al., 2005; Jiang et al., 2006; Wierzbicki et al., 2013). Loss of heterozygosity in the *LATS1* chromosomal region was reported in ovarian tumors, breast tumors and adenoid cystic carcinoma (Hansen et al., 2002; Takahashi et al., 2005; Rutherford et al., 2006).

The human *LATS1* gene is 57.3 Kb in length and is located at chromosome 6q24–25.1 (Nishiyama et al., 1999). Large-scale genome sequencing studies have identified various mutations within this chromosomal region (Koed et al., 2005). Chromosome 6 was shown to be a region that is frequently subjected to allelic imbalances in bladder tumors (Koed et al., 2005). High heterozygosity regions and nucleotide diversity were observed on chromosome 6 and a total of 96,317 SNPs were mapped to chromosome 6, with SNPs density about a single SNP every 3.07 Kb (Sachidanandam et al., 2001). Particular SNPs in this region were correlated with certain diseases. For instance, a genome-wide association study (GWAS) of 19,091 cases with breast cancer and 20,606 controls has identified the SNP rs9485372 in chromosome 6q25.1 near the *LATS1* gene, and associated it with risk to breast cancer (Long et al., 2012).

The genomic instability of the *LATS1* chromosomal region and the LATS1 down-regulation that was associated with various types of tumors intrigued a study of SNPs and insertions/deletions (indels) within the *LATS1* gene, as well as to assess the effect of these single-base-sequence alterations on LATS1 expression in urinary bladder. Because of regional prevalence of urinary bladder and colon cancer and the latter has been previously reported to be associated with LATS1 promoter hypermethylation (Wierzbicki et al., 2013), we selected these particular tissues. The aim of this study was achieved by scanning genomic *LATS1* for SNPs and indels in urinary bladder and colon tissues from Egyptian patients. A total of 29 variants were identified, including 18 novel variants and 11 SNPs previously identified in the *LATS1* gene (NCBI dbSNP Human Build 141, Released: 05/21/2014)^1^.

## Materials and methods

### Patients and tissue samples

Tissue samples were collected from 28 patients (25 males and 3 females). Informed consent to use tissue excised from patients by the Pathology Department of Cairo University for the current study was obtained from the 28 patients' who voluntarily joined the study. Data of the collected specimen were analyzed anonymously. The Institutional Review Board (IRB) of the National Cancer Institute and the IRB of the American University in Cairo approved all research protocols, followed in this study. The patients mean age were 56.6 ± 12.8 (S.D) years. Based on patients histopathology records, the 28 patients were categorized into 4 groups; 3 normal urinary bladder tissues, 25 urinary bladder cancer tissues, 1 normal colon tissue and 3 Colon cancer tissues. The normal tissues were excised from the tissue surrounding tumor tissue and were used as control. All the 28 samples were used for SNPs detection and assessing microindels (≤10 nucleotides).

### RNA extraction, cDNA synthesis, and PCR for checking LATS1 expression

Total RNA was extracted from tissues using Trizol reagent. DNase digestions were done for all samples using RQ1 RNase-Free DNase enzyme (Promega) to eliminate DNA contamination. One-step cDNA were performed using Access RT-PCR system (Promega) and GAPDH amplifications were performed to ensure that the extracted RNA was sufficient for PCR amplification following DNase digestion. This was followed by a two steps cDNA, using RevertAid H Minus First Strand cDNA Synthesis Kit (Fermentas), using random hexamer primers.

PCR was performed and the RNA steady state levels of *LATS*1 were normalized to GAPDH. Forward and reverse PCR primers were LATS1 Forward primer within exon 1, 5′- GCCTGGTGTTAAGGGGAGAG-3′, and reverse primer within exon 2, 5′- CAAGTCTTGAAGCATTTGTGGA-3′, and GAPDH Forward primer, 5′-TTAGCACCCCTGGCCAAGG-3′, and reverse primer, 5′CTTACTCCTTGGAGGCCATG-3′ (Lin et al., 2000). Expected PCR products sizes are LATS1 660 bp and GAPDH 541 bp.

### DNA extraction and PCR amplification

**Figure 1:**
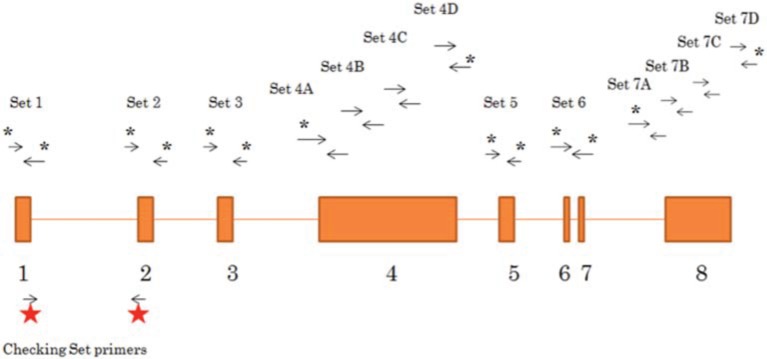
**Schematic representation of primers sets used to amplify the *LATS1* gene**. 13 sets of primers were designed (sequence details shown in Supplemental Table 1) to amplify and sequence *LATS1*. Primer sets with an asterisk are the sets used for amplification and sequencing of the *LATS1* 8 exon and the adjacent 5′ and 3′ intronic region. The sets without an asterisk are used in the sequencing only. The primer sets used to assess *LATS1* expression are presented with the red stars.

Genomic DNA was extracted from all tissue samples using the DNeasy Blood & Tissue Kit (QIAGEN). Seven sets of primers were used to amplify the exons and the adjacent intronic region (~7000 bp). The primers utilized and the regions amplified are illustrated in Figure 1 and supplemental Table 1. Multiple PCR amplifications were done using FastStart High Fidelity PCR System (5 U/ aphic data μL) (ROCHE). The PCR products were resolved on 1% agarose gels and purified using QIA quick Gel Extraction Kit (QIAGEN).

### Restriction fragment length polymorphism (RFLP) for PCR amplicons

Prior to sequencing PCR products, RFLP was performed to confirm the *LATS1* PCR amplified product. We used restriction digestion enzymes that didn't overlap with reported mutations retrieved from the NCBI *LATS1* SNPs database. TAQαI, EcoRI, EcoRV, and EcoRI were used on PCR products of primer sets 1, 3, 5, and 6, respectively. DpnI was used with primer set 6 PCR amplicons that failed to digest with EcoRI, because DpnI restriction enzyme cuts fully-adenomethylated DNA. Primer sets 2, 4, and 8 PCR amplicons were directly sequenced. Products confirmed by RFLP (data not shown) were sequenced.

### Sequencing

Sequencing was done using the BigDye Terminator v3.1 Cycle Sequencing Kit from Applied Biosystems. Each fragment from the 13 amplified portions of each sample (28 samples) was sequenced at least twice (using the forward and reverse primers). The sequences generated by the same primer pairs were assembled for each of the four-histopathology patient group. This would allow us to confirm single-base-sequence alterations in the sequence, from the Forward and Reverse sequence.

### Data analysis, analysis software: Polyphred

The *LATS1* sequences of each sample, in each of the 4 groups; 1- Urinary Bladder Cancer, 2- normal peritumoral urinary Bladder, 3- colon cancer, and 4- normal peritumoral colon were assembled. Each group of sequences was aligned together and analyzed using PolyPhred software program Version 6.15 Beta. Only SNPs of rank 1 (97% for true positive SNP) and rank 2 (75% for true positive SNP) were considered. All SNPs identified by PolyPhred were compared to the NCBI *LATS*1 SNP database (Sherry et al., 2001). SNP identified in our study and match the NCBI *LATS*1 SNPs (NCBI dbSNP Human Build 141, Released: 05/21/2014) were considered an old SNP and those not matching the SNP database were considered new variants.

Codon found to have SNPs were checked manually for the amino acid it encodes and compared to the wild type version. single-base-sequence alterations were checked for biological significance to determine overlap with the Serine/ Threonine kinases catalytic domain. Additionally small indels (insertions/ deletions) of length 10 base pairs or less were analyzed using PolyPhred.

### Bioinformatic analysis

TRANSFAC Database Match tool (Kel et al., 2003; Matys et al., 2006) was used to annotate the potential transcription factor (TF) binding sites within the *LATS1* gene using the positional weight matrices database derived from the experimentally-proven binding sites. Genome Trax™ (www.biobase-international.com/genome-trax) analysis from BIOBASE Corporation was used to map the SNPs/variants to the potential regulatory regions in the *LATS1* gene. Softberry-SNP-effect1 (Softberry Inc., www.softberry.com) was used to predict the potential impact of the SNPs/variants on the protein structure.

## Results

### LATS1 amplification and sequencing

Peritumoral urinary bladder tissues, urinary bladder cancer tissues, peritumoral colon tissue and colon cancer tissues were collected from 28 patients suffering from urinary bladder cancer or colon cancer. Table 1 summarizes patients' clinical characteristics. The genomic DNA from the tissues were extracted, fragments of *LATS1* were amplified and sequenced followed by analysis of SNPs and indels and microindels. Primers used to amplify the *LATS*1 gene are listed in supplemental Table 1 and presented in Figure 1. RFLP was utilized to confirm the amplicons of exons 1, 3, 5, 6, and 7 prior to sequencing (Supplemental Table 2). Alignment of *LATS1* amplicon/sequences to previously reported *LATS*1 gene was performed and all *LATS*1 amplicon/sequences aligned to their respective position.

**Table 1:** **The clinical characteristics of patients in the study**.

**Variables**	**Peritumoral urinary bladder cases**	**Urinary bladder cancer**	**Peritumoral colon cases**	**Colon cancer cases**
Total number	3	25	1	3
Male\female	3\0	23\2	1\0	2\1
Mean age (years)	NA^*^	56.6 ± 12.8	NA^*^	NA^*^
Smoking	2\1	8\15	NA^*^	NA^*^
Bilharziasis	1\2	3\22	NA^*^	0\3

* *NA, Not Available*.

### Identification of the LATS1 variants including single nucleotide polymorphisms (SNPs)

A total of 29 SNPs/variants were identified in the *LATS1* gene. Among these 29 SNPs, 18 were novel variants and 11 SNPs were previously identified in the *LATS1* gene in earlier studies (Table 2). Seven SNPs/variants were identified in the coding region of the *LATS1* gene (Table 2). While, the other 22 SNPs/variants were in the non-coding region of the gene, including 5′UTR, introns and 3′UTR. Interestingly certain SNPs/variants were associated with a particular type of tissue; two variants were only found in the *LATS1* gene of the urinary bladder cancer tissues, six peritumoral specific variants were found in the *LATS1* of the peritumoral urinary bladder tissues, another six peritumoral specific SNPs/variants were found only in the peritumoral colon cancer tissues and four variants were identified in the colon cancer tissues (Table 2). Other SNPs/variants were not tissue-specific, and were common between different tissue types. For instance, 4 SNPs were identified in the urinary bladder cancer as well as peritumoral urinary bladder tissues and 5 SNPs/variants were identified in the peritumoral and cancerous colon tissues.

**Table 2:** **Summary of the SNPs/variants detected in the LATS1 gene**.

**Consensus position**	**The variant/SNP genomic position**	**Position of the SNP/variants**	**SNP/variant type**	**Novelty**	**Corresponding amino acid change**	**Tissue samples with the SNP/variant**
32	150039361	5′UTR (exon 1)	G > C	Previously identified	−	CC1, CC2, CC3, and NC1
100	150039293	5′UTR (exon 1)	A > G	Previously identified	−	NC1
245	150039148	5′UTR (exon 1)	C > T	Previously identified	−	UN1^*^, UN2, and CC1, CC2^***^, CC3, NC1^**^
342	150039051	5′UTR (exon 1)	T > A	Novel	−	CC2
439	150038954	5′UTR (exon 1)	T > A	Novel	−	CC1, CC2, CC3
450	150038943	5′UTR (exon 1)	T > A	Novel	−	CC2
515	150038878	5′UTR (exon 1)	T > A	Novel	−	UN1, UN2, UN3
15908	150023485	Intron 1	C > A	Novel	−	UN2
15943	150023450	Intron 1	T > A	Novel	−	UN2, UN3
16045	150023348	5′UTR (exon 2)	G > C	Previously identified	−	UC1^****^, UC9, UC11, UC13, UC15, UC19, UC20, UC23, and NC1
16283	150023110	Exon 2, coding region	G > A	Novel	Glu51Glu	NC1
22891	150016502	Intron 2	T > C	Previously identified	−	NC1
22899	150016494	Intron 2	G > C	Novel	−	CC2, CC3, and NC1
34614	150004779	Exon 4, coding region	T > C	Previously identified	Ser482Ser	UC1, UC4, UC5, UC9, UC11, UC15, UC16, UC21, UC23, and UN1, UN2
35366	150004027	Intron 4	T > G	Previously identified	−	UC1, UC4, UC8, UC12, UC13, UC14, UC15, UC20, UC21, UC23, and UN1
38335	150001058	Exon 5, coding region	T > A	Novel	Leu849His (Serine/Threonine catalytic domain)	UN1
41346	149998047	Intron 6	G > A	Novel	−	CC1, CC2, CC3
41625	149997768	Exon 6, coding region	G > A	Novel	Arg900His (Serine/Threonine catalytic domain)	UC1, UC4, UC5, UC8, UC9, UC12, UC16, UC17, UC18, UC23, UC24
41736	149997657	Intron 6	A > G	Previously identified	−	UC1, UC4, UC5, UC7, UC8, UC9, UC10, UC11, UC12, UC13, UC15, UC16, UC17, UC20, UC21, UC23, UC24, and UN2, UN3
42029	149997364	Intron 6	T > A	Novel	−	UC5, UC8, UC10, UC12, UC13, UC15, UC21, UC23, UC24
56152	149983241	Exon 8, coding region	C > T	Novel	Ala1006Val (Serine/Threonine catalytic domain)	UN2, UN3
56164	149983229	Exon 8, coding region	T > C	Novel	Phe1010Ser (Serine/Threonine catalytic domain)	UN2, UN3
56177	149983216	Exon 8, coding region	C > T	Previously identified	Asp1014Asp	UC1, UC8, UC9, UC11, UC15, UC21, UC23, and UN1, UN3, NC1
56976	149982417	Exon 8, 3′UTR	T > C	Previously identified	−	NC1
57142	149982251	Exon 8, 3′UTR	A > C	Previously identified	−	CC1, CC2, CC3, and NC1
57149	149982244	Exon 8, 3′UTR	G > C	Novel	−	CC1, CC2, CC3, and NC1
57154	149982239	Exon 8, 3′UTR	A > C	Novel	−	NC1
57155	149982238	Exon 8, 3′UTR	T > A	Novel	−	CC1, CC2, CC3, and NC1
57160	149982233	Exon 8, 3′UTR	T > A	Novel	−	NC1

* UN, peritumoral urinary bladder tissue;

** NC, peritumoral colon tissue;

*** CC, colon cancer tissue;

**** *UC, urinary bladder cancer tissue*.

The seven SNPs/variants identified in the coding region of *LATS1*, comprise 3 SNPs/variants encoding silent mutations and 4 variants encoding missense mutations (Table 2). The missense variants result in alteration of 4 different amino acids within the Serine/Threonine catalytic domain of the LATS1 protein. The novel variant at consensus position 38335, within exon 5, was identified only in a peritumoral urinary bladder tissue (UN1). This variant resulted in the replacement of leucine amino acid with a histidine amino acid, at amino acid position 849, within the Serine/Threonine catalytic domain. On the other hand, the novel variant at consensus position 41625, located at exon 6 of the *LATS1* gene, was only detected in urinary bladder cancer tissues. This resulted in alteration of an arginine amino acid into histidine at the amino acid position 900, located at the Serine/Threonine catalytic domain of the LATS1 protein. The peritumoral urinary bladder tissues showed another two novel variants at exon 8 that resulted in two missense mutations within the LATS1 protein. These Serine/Threonine catalytic domain variants mutates alanine to valine at amino acid position 1006, and phenylalanine to serine at amino acid position 1010 (Table 2).

The three SNPs/variants within the coding region that caused silent mutations includes a novel variant found in the peritumoral colon tissue at consensus 16283 in exon 2 (G > A) and encodes for glutamic acid (Table 2). The two other silent SNPs include a previously identified SNP at exon 4, detected in urinary bladder cancer and peritumoral urinary bladder tissues, and a previously identified SNP at exon 8 detected in the cancerous, peritumoral urinary bladder tissues as well as peritumoral colon tissue. These single-base-sequence alterations at consensus 34614 in exon 4 (T > C) and consensus 56177 in exon 8 (C > T) encode for serine and aspartic acid, respectively (Table 2).

Majority of the 22 SNPs/variants identified within the non-coding region of the *LATS1* gene were located in both the 5′ and 3′UTRs. There were 8 SNPs/variants detected within exon 1 and 2 that corresponds to the 5′ UTR region, 6 SNPs/variants within the 3′UTR within exon 8 and 8 SNPs/variants within the introns 1, 2, 4, and 6 of the *LATS1* gene (Table 2). Among the eight SNPs/variants at the 5′UTR, 3 newly identified variants were only detected in the colon cancer tissues (Table 2). Of the eight SNPs/variants identified in the intronic regions (Table 2), only three SNPs were previously reported. Two SNPs located within intron 1 of the *LATS1* gene were only detected in the peritumoral urinary bladder tissues. Five new variants were found in the 3′UTR region (exon 8) and were only detected in colon cancer and peritumoral colon tissue groups suggesting that they could be tissue specific.

### Sequence transposition within the LATS1 gene

Primer set 5P (shown in Supplemental Table 1), designed to align to exon 4 at consensus position 34481–34965 on the genomic DNA, aligned to intron 5 to intron 7 of the *LATS1* gene in one of the three examined colon cancer tissues. This suggests that in this sample exon 4 of the *LATS1* gene was shifted to a new position between intron 5 to intron 7 of the *LATS1* gene in this colon cancer sample. Note that the remaining 25 urinary bladder cancer tissues and two colon cancer tissues amplified the intended exonic region.

### The expression of LATS1 gene harboring different SNPs/variants

**Figure 2:**
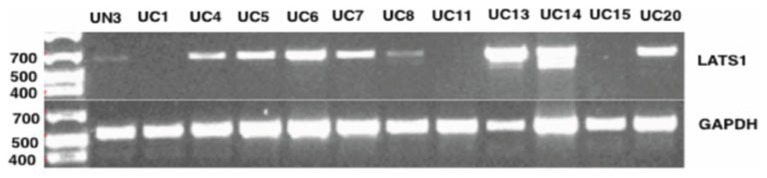
***LATS1* expressions analysis using PCR**. LATS1 expression was examined in urinary bladder cancer tissues (UC), peritumoral urinary bladder (UN). UC6 did not contain any SNP in the LATS1 gene. UC7 showed SNP at 41736; UC14 harbors SNP at 35366; UC1 has SNPs/variants at 16045, 34614, 35366, 41625, 41736, and 56177; UC4 has SNPs/variants at 34614, 35366, 41625, and 41736; UC5 has SNPs/variants at 34614, 41625, 41736, and 42029; UC8 has SNPs/variants at 35366, 41625, 41736, 42029, and 56177; UC11 has SNPs at 16045, 34614, 41736, and 56177; UC13 harbors SNPs/variants at 16045, 35366, 41736, and 42029; UC 15 harbors SNPs/variants at 16045, 34614, 35366, 41736, 42029, and 56177; UC 20 harbors SNPs at 16045, 35366, and 41736. UN3 harbors SNPs/variants at 515, 15943, 41736, 56177, 56152, and 56164. GAPDH was used as an endogenous control. Generuler 1 Kb Plus DNA Ladder (Fermentas®) was used as a size marker.

To assess if the various SNPs/variants alter the expression of the *LATS1* gene, we examined the RNA steady state levels of *LATS1* in the different urinary bladder cancer tissues utilized in this study (Figure 2). The *LATS1* gene was differentially expressed in the different tissues. A urinary bladder cancer tissue sample (UC13) showed higher levels of the *LATS1* expression compared with peritumoral tissue and urinary bladder cancer tissue without any variation within the LATS1 gene (UC6) (Figure 2). Nevertheless, *LATS1* RNA steady state levels were not observed in 3 urinary bladder cancer tissue samples (UC1, UC11, and UC15). The urinary bladder tissue samples examined, comprises *LATS1* gene with different SNPs/variants, showed different levels of *LATS1* gene expression.

A total of seven SNPs/variants were identified in the urinary bladder cancerous tissues (Table 3). All of these SNPs/variants have a representative sample that was examined for the *LATS1* expression. The urinary bladder cancerous tissue (UC6), that did not comprise any variation within the *LATS1* gene, expressed *LATS1*. Three urinary bladder cancerous tissues (UC1, 11, and 15) did not express the *LATS1* gene. These three samples were the only samples that contained the combination of four previously identified SNPs at positions 16045, 34614, 41736, and 56177. The SNPs at consensus position 16045 and 41736 are located within the 5′UTR and intron 6, respectively. The other two SNPs (positions 34614 and 56177) represented missense mutations located at exon 4 and exon 8, respectively.

**Table 3:** **Summary of the SNPs/variants detected in the *LATS1* of the urinary bladder cancerous tissues**.

**Samples**	**No. of SNPs/variants within the LATS1 gene of this sample**	**Position of SNP/variant**	**LATS1 expression**
UC2, UC3, UC6, UC22, UC25	No SNPs		U6 showed + LATS1 expression.
UC19	1 SNP	16045	NA
UC14	1 SNP	35366	+
UC18	1 variant	41625	NA^**^
UC7	1 SNP	41736	+^*^
UC10	2 SNPs/variants	41736 + 42029	NA
UC17	2 SNPs/variants	41625 + 41736	NA
UC20	3 SNPs	16045 + 35366 + 41736	+
UC16	3 SNPs/variants	34614 + 41625 + 41736	NA
UC24	3 SNPs/variants	41625 + 41736 + 42029	NA
UC13	4 SNPs/variants	16045 + 35366 + 41736 + 42029	+++
UC11	4 SNPs	**16045** + **34614** + **41736** + **56177**	−^***^
UC4	4 SNPs/variants	34614 + 35366 + 41625 + 41736	+
UC5	4 SNPS/variants	34614 + 41625 + 41736 + 42029	+
UC12	4 SNPs/variants	35366 + 41625 + 41736 + 42029	NA
UC9	5 SNPs/variants	16045 + 34614 + 41625 + 41736 + 56177	NA
UC21	5 SNPs/variants	34614 + 35366 + 41736 + 42029 + 56177	NA
UC8	5 SNPs/variants	35366 + 41625 + 41736 + 42029 + 56177	±
UC1	6 SNPs/variants	**16045** + **34614** + 35366 + 41625 + **41736** + **56177**	−
UC15	6 SNPs/variants	**16045** + **34614** + 35366 + **41736** + 42029 + **56177**	−
UC23	7 SNPs/variants	16045 + 34614 + 35366 + 41625 + 41736 + 42029 + 56177	NA

* *+, Showed LATS1 mRNA expression*.

** *NA, Not Available*.

*** *−, Did not show LATS1 mRNA expression*.

*The bold font indicates the position of four SNPs, when combined, were associated with eliminated steady state levels of LATS1 RNA*.

### Identification of the potential regulatory regions within the LATS1 gene

The differential expression of the *LATS1* mRNA in the urinary bladder cancer tissues that shared *LATS1* SNPs/variants suggested a role for the SNPs/variants (in non-coding regions) in *LATS1* expression. *In silico* analysis of the *LATS1* sequence using Transfac database identified TF binding including GEN_INI, AP-2alphaA, ZNF263, p53, ZF5, CDP CR1, and Nanog, that overlap with five SNPs/variants (5′UTR), located at positions 100, 342, 439, 450, and 16045 (Table 4). Using Genome Trax to map the SNPs/variants to potential regulatory regions in the *LATS1* sequence revealed CpG_islands that overlap with seven SNPs/variants (5′UTR) (Table 4). The position of other non-coding SNPs/variants at intron-2, 4, and 6 as well as at 3′UTR overlap with potential binding sites for several TFs including FAC1, GR, HNF-3beta, ardi5a, Zfp105, RBP-Jkappa, Xvent-1, RREB-1, Sp1, RREB-1, GKLF, Churchill, GKLF, CPBP, SF-1, Nanog, RUSH-1alpha, SOX10, and SRY (Table 4).

**Table 4:** **The reported LATS1 SNPs/variants that were mapped to the TF-binding regions predicted by Transfac database (Kel et al., 2003; Matys et al., 2006)**.

**SNP/variant consensus position**	**SNP/variant position**	**CpG_island**	**TF type**	**TFBS consensus sequence within LATS1**	**Mutated sequence**
32	5′UTR (exon 1)	CpG_island			
100	5′UTR (exon 1)	CpG_island	GEN_INI	(96) cccC**A**GTC	cccC**g**GTC
245	5′UTR (exon 1)	CpG_island			
342	5′UTR (exon 1)	CpG_island	AP-2alphaA	(336) acGGAC**T**ctggccgc	acGGAC**a**ctggccgc
439	5′UTR (exon 1)	CpG_island	ZNF263	(438) c**t**CCTCC	c**a**CCTCC
450	5′UTR (exon 1)	CpG_island	p53	(446) gtgC**T**CGAgttggcggggcg	gtgC**a**CGAgttggcggggcg
			ZF5	(441) ctccagtGC**T**CGag	ctccagtGC**a**CGag
515	5′UTR (exon 1)	CpG_island			
15943	Intron 1		Myogenin	(15942) C**t**gCTG	C**A**gCTG
16045	5′UTR (exon 2)		CDP CR1	(16043) ag**g**aTCATTt	ag**c**aTCATTt
			Nanog	(16043) ag**g**aTCATTttc	ag**c**aTCATTttc
22891	Intron 2		HNF-3beta	(22891) **t**acAAACAg	**c**acAAACAg
22899	Intron 2		FAC1	(22894) aaacA**G**AACattaa	aaacA**c**AACattaa
			GR	(22897) cA**G**AACattaatatca	cA**c**AACattaatatca
35366	Intron 4		ardi5a	(35362) tcAA**T**ATttattca	tcAA**g**ATttattca
			Zfp105	(35357) gtattTCAA**T**atttatt	gtattTCAA**G**atttatt
41346	Intron 6		Xvent-1	(41344) ca**g**AAAATaggct	ca**a**AAAATaggct
42029	Intron 6		SREBP	(42023) gaatTC**t**CACAaa	gaatTC**A**CACAaa
			RBP-Jkappa	(42025) atTC**T**CAcaaa	atTC**A**CAcaaa
56976	Exon 8, 3′UTR		SF-1	(56966) ctcataaCCT**T**Gtt	ctcataaCCT**c**Gtt
			Nanog	(56967) tcataacCT**T**GTttttggta	tcataacCT**c**GTttttggta
			RUSH-1alpha	(56971) aacCT**T**GTtt	aacCT**c**GTtt
			FAC1	(56972) acct**t**GTTTTtggt	acct**c**GTTTTtggt
			SOX10	(56973) cCT**T**GTt	cCT**c**GTt
			SRY	(56975) T**T**GTTt	T**c**GTTt
57142	Exon 8, 3′UTR		RREB-1	(57137) cCCCC**A**ccccccgc	cCCCC**c**ccccccgccc
			Sp1	(57137) cccCC**A**CCcc	cCC**c**CCcc
			RREB-1	(57138) cCCC**A**Ccccccgcc	cCCC**c**Ccccccgcc
			GKLF	(57140) CC**A**CCcc	CC**c**CCcc
57149	Exon 8, 3′UTR		Churchill	(57144) cCCCC**G**	cCCCC**c**
			Sp1	(57144) cccCC**G**CCcc	cccCC**c**CCcc
			GKLF	(57147) CC**G**CCcc	CC**c**CCcc
			CPBP	(57149) **G**CCCCat	**c**CCCCat

*Bold letters indicate locations of the variants*.

*The upper case letters indicate highly conserved core binding sequence of the TFs*.

To predict the effect of the SNPs/variants, located at the coding regions, on the LATS1 protein structure, we used Softberry-SNP-effect *in silico* tool to assess the impact of amino-acid alterations. Four missense mutations and a single silent mutation were reported in the serine-threonine catalytic domain. These five single-base-sequence alterations, which are Leu849His, Arg900His, Ala1006Val, Phe1010Ser, and Asp1014Asp, were predicted to produce a damaged protein. On the other hand, the two silent mutations, Glu51Glu, and Ser482Ser, that are located at LATS Conserved Domain-1 and LATS Conserved Domain-2, respectively, were predicted to be tolerated sequences for the protein.

## Discussion

The large tumor suppressor 1 (*LATS1*) is inactivated in various tumor types encompassing soft tissue sarcomas, and adenoid cystic carcinoma, as well as, breast, lung, prostate and esophageal cancers. The inactivation of the *LATS1* could be initiated by allelic loss of heteozygosity, gene deletion, point mutation or hyper-methylation of the promoter region. However, *LATS1* mutations and biological significance in cancers remain unidentified. This study examines the genomic *LATS1* region for single-base-sequence alterations in urinary bladder and colon cancer tissues.

We have therefore designed 13 primer sets to amplify and sequence the genomic *LATS1* fragments including the eight exons of the *LATS1* gene, the adjacent intronic region, the exon-intron boundaries, the 5′UTR and the 3′UTR (Figure 1). This allowed us to screen for single-base-sequence alterations that could disrupt the regulatory regions, the alternative splicing sites or the coding regions in urinary bladder and colon tumors. The sequenced 13 amplified fragments of the 28 tissue samples were assembled and compared to the *LATS1* sequence on the NCBI (NC_000006.1). We detected 29 variants/SNPs within the *LATS1* gene in urinary bladder and colon tumors, including 18 novel variants and 11 previously identified SNPs (Table 2). Certain SNPs/variants were specifically detected in either urinary bladder or colon tissues and other were detected in both tissue types.

Among these 29 SNPs/variants, there were 22 SNPs/variants within the non-coding region (introns and UTRs) and seven SNPs/variants located within the coding region of the *LATS1* gene including three silent and four missense SNPs/variants. All the reported missense variants are located within the Serine/Threonine catalytic domain of the LATS1 protein (Table 2). These four missense variants altered four amino acids at amino acid position L849H, R900H, at A1006V and at F1010S within the Serine/Threonine catalytic domain at amino acid position 708–1130 on the *LATS1* protein (Table 2). The missense mutation L849H was also detected in stomach cancer (data not shown). These variants are likely to influence the activity of the tumor suppressor gene *LATS1* through changing the amino acid sequence of its' catalytic domain. Although the functionality of the *LATS1* protein with the genotyped SNPs/variants remains unidentified, the results reveal an association of these missense variants with the peri-tumoral and tumor tissues that may impact the tumor suppressor activity of LATS1. The other three SNPs/variants at the exons region of exon 2 (A/G), exon 4 (C/T), and exon 8 (T/C) were silent and did not alter the amino acid sequence of the encoded LATS1 protein.

We identified 22 SNPs/variants at the *LATS1* non-coding region including eight at the 5′UTR, six at the 3′UTR, and eight at intronic regions. We scanned the intron-exon boundaries and the possible SNPs/variants that may affect the alternative splicing. However, none of the reported SNPs/variants were located at splice sites. SNPs/variants at non-coding regions may alter *LATS1* expression and affect the consequent post-transcription regulation of the downstream genes. These SNPs/variants could have interrupted an essential regulatory region of *LATS1*. Therefore, we examined the RNA steady state levels of *LATS1* in the urinary bladder cancer tissues as representatives of different SNPs/variants within the *LATS1* gene. However, further studies are desirable to analyze the consequences of the *LATS1* non-coding SNPs/variants on the *LATS1* activity. That being said, in the present study we observe an association of these SNPs/variants with the differential expression of LATS1 in urinary bladder tumors (Figure 2).

Moreover, a transposition of exon 4 into another position at intron 5 to intron 7 of the *LATS1* gene was observed in a colon cancer tissue. This transposition within the *LATS1* gene alters the amino acid sequence of the Serine/Threonine catalytic domain of *LATS1* protein and may result in *LATS1* inactivation in colon cancer. The steady state levels of *LATS1* RNA was examined using PCR primers that cover the region from second half of exon 1 to first half of exon 2, which allows detection of the two isoforms of the *LATS1* mRNA (Figure 1). One limitation in this study is the lack of information on *LATS1* expression levels for the entire cohort. However, a total of seven different SNPs/variants were reported in urinary bladder cancerous tissues and all of them had representative tissue that was examined for the RNA steady state levels of *LATS1* (Table 3). A urinary bladder cancerous tissue (UC6) that did not encompass any SNPs/variants showed *LATS1* expression (Figure 2). On the other hand, the urinary bladder cancerous tissues (UC1, 11, and 15) that shared SNPs at 16045, 34614, 41736, and 56177, appear to have similar *LATS1*-gene-expression-profile alteration (Figure 2). The results suggested that these combined SNPs likely contribute to the disruption of *LATS1* RNA steady state levels. On the other hand, the urinary bladder cancer tissue (UC13) that encompass four SNPs/variants at 16045, 35366, 41736, and 42029, which are located at the 5′UTR and introns regions, showed high expression levels of the *LATS1* gene compared to the urinary bladder cancer tissue without *LATS1* SNPs/variants (UC6). The urinary bladder cancerous tissue (UC8) showed remarkably low levels of *LATS1* mRNA (Figure 2). Five SNPs/variants were reported within the *LATS1* of UC8 at position 35366, 41625, 41736, 42029, and 56177, resulting in three SNPs/variants in the introns 4 and 6, besides a silent SNP within exon 8 and a missense variant within exon 6 that encodes for the catalytic domain. Moreover, we observed that the examined samples that encompass the SNP at position 56177 within exon 8 of *LATS1*, which resulted in silent mutation in the *LATS1* catalytic domain, showed very low or no *LATS1* expression, including the peritumoral urinary bladder tissue UN3 as well as the urinary bladder cancerous tissues UC1, UC8, UC11, and UC15. These observations emphasize the impact of each single single-base-sequence alterations may have on the tumor suppressor *LATS1* gene expression in cancer, as well as the contribution of these SNPs/variants in *LATS1* inactivation and the subsequent increased risk of urinary bladder and colon malignancies.

**Figure 3:**
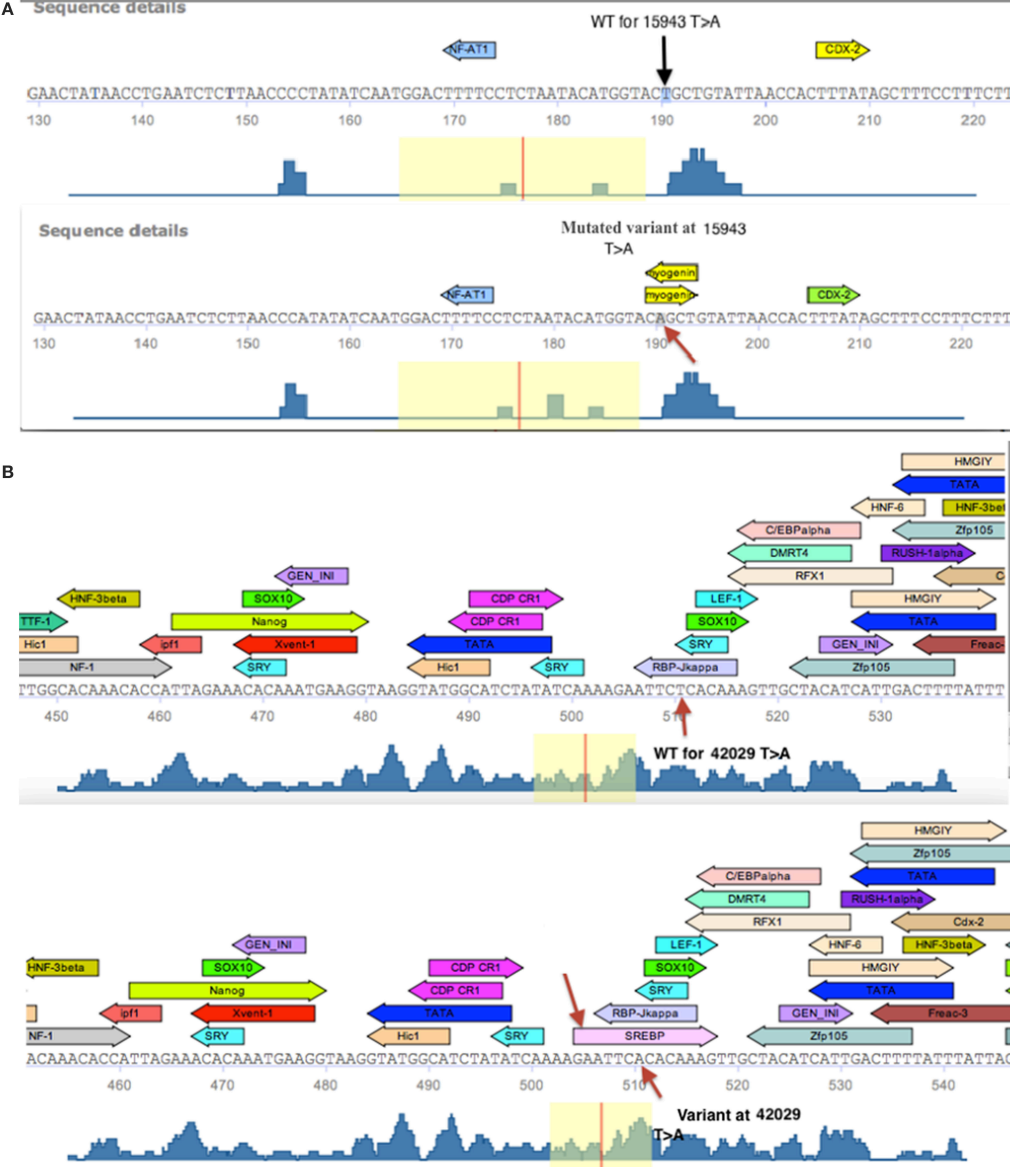
**Schematic diagram of the Transfac predicted TF binding sites within a fragment of *LATS1* gene. (A)** Transfac diagram of the wild type (WT) fragment of *LATS1* intron-1 that depicts the myogenin transcription factor-binding site as created by the variant at consensus position 15943. **(B)** Diagram of the depicted SREBP TF binding site that is generated by the variant at 42029 (indicated by an arrow) at LATS1 intron-6 as well as the predicted TF binding sites that were interrupted by the variant (Kel et al., 2003; Matys et al., 2006).

The differential expression of the *LATS1* mRNA in the urinary bladder cancer tissues that shared *LATS1* SNPs/variants urged us to assess potential roles for SNPs/variants in the non-coding regions of *LATS1* on its expression. *In silico* analysis of the *LATS1* sequence using Transfac database revealed that the SNPs/variants interrupt several consensus TFs binding sites (Table 4). Such SNPs/variants may disrupt the binding of the cognate TF. TRANSFAC identified five SNPs/variants to overlap with predicted binding sites of various TFs. Genome Trax identified CpG_islands, which were mapped to seven SNPs/variants at the 5′UTR (Table 4). The analysis identified SNPs/variants that disrupted the TF recognition sequence of one TF but maintained the binding of another TF. For instance the SNP at 35366, allowed Zfp105 TF binding but resulted in loss of the ardi5a binding site, a TF that stimulates histone acetylation. The variant at 15943, which is located within *LATS1* intron 1, did not interrupt a regulatory region. However, this mutation generated the core sequence (C**A**gCTG) for the binding of Myogenin TF, which is a TF that regulates cell cycle and stem cell proliferation and was associated with various types of tumors including rhabdo-myosarcoma, liver neoplasms, soft tissues neoplasms, and neuroectodermal tumors (Figure 3A) (Wang et al., 1995; Meyer and Brinck, 1997; Kumar et al., 2000; Kobayashi et al., 2004). Likewise, a variant at intron 6, located at consensus position 42029, created the consensus sequence for the binding of the TF SREBP (Figure 3B). The effect of this variant could have contributed to *LATS1* over-expression observed with the urinary bladder cancer tissue (UC13), at which the variant at 42029 was reported. Therefore, such SNPs/variants may be the aberrant building blocks that disrupt cellular mechanisms and contribute to the urinary bladder and colon malignances.

We report alterations of single base pairs in the *LATS1* gene, of Egyptian patients with urinary bladder and colon cancer tissues. Additionally, we identified novel variants within the *LATS1* gene that contributed to the down regulation of its mRNA expression in urinary bladder tissues. *LATS1* variants and its' relationship to ethnicity hasn't been previously addressed. Therefore, we cannot deduce that the novel variants identified would be different from other racial populations. However, this study reveals the significance of the tumor suppressor activity of LATS1 and highlights the association of *LATS1* single-base-sequence alterations with urinary bladder and colon tumors. Nonetheless, further studies need to be conducted with relatively larger sample size and evaluate the functionality of the LATS1 protein with the detected SNPs/variants.

## Author contributions

Mona K. Saadeldin carried out the molecular studies, data analyses and participated in drafting the manuscript. Heba Shawer carried out the proteomic studies, participated in data analyses and drafting the manuscript. Ahmed Mostafa provided/collected specimens that were used to accumulate data. Neemat M. Kassem participated in the experimental design. Rania Siam and Asma Amleh conceived the study, participated in its design, data analyses and drafted the manuscript to a publishable form. All authors read and approved the final manuscript.

### Conflict of interest statement

The authors declare that the research was conducted in the absence of any commercial or financial relationships that could be construed as a potential conflict of interest.

## Footnotes

^1^Database of Single Nucleotide Polymorphisms (dbSNP). Bethesda, MD: National Center for Biotechnology Information, National Library of Medicine. Available online at: http://www.ncbi.nlm.nih.gov/SNP/

## Abbreviations

SNPs: single nucleotide polymorphisms
LATS: large tumor suppressor gene
MST1/2: mammalian Ste20-like serine/threonine kinases 1 and 2
YAP: yes-associated protein
BAX: Bcl-2-associated X
GWAS: genome-wide association study; Indels: insertions/deletions
UN: urinary bladder peritumoral tissue
NC: peritumoral colon tissue
CC: colon cancer tissue
UC: urinary bladder cancer tissue
CDC2: Cell Division Control
TF: transcription factor.
