# Supplementary material for: New genetic variants of LATS1 detected in urinary bladder and colon cancer
Source: Front Genet. 2015 Jan 13;5:425. doi: 10.3389/fgene.2014.00425 (PMC4292772; doi:10.3389/fgene.2014.00425)
Supplement: Supplementary file 1 [file DataSheet1.DOCX]

1. **Supplementary Material**

**Supplemental Table 1. Primer sets used for genotyping SNPs/variants of LATS1**

| Primer | Forward Primer sequence (5’→3’) | Reverse Primer sequence (5’→3’) | Product size | Annealing sites | LATS1 Region |
| --- | --- | --- | --- | --- | --- |
| SET 1 | GCGACGCTCACGAACGATCA | CGGGTCAATGCAACCAGCCA | 571 | 1-573 | 1 |
| SET 2 | AGGCTAGGAAGTAGGCATGGAG | CCCAAAGGAAACATTTTGGCATGT | 759 | 15813-16571 | 2 |
| SET 3 | TGCGCCCTGCCTGTTTTAGT | GTCTGAATGATGGGATTTGGAGGGA | 632 | 22843-23474 | 3 |
| SET 4A1 | CTCTCGGCAATTTTCAGGAGTACAA | GCTAGAACTCTGCATGATGATTGGT | 720 | 33457-34176 | 4 |
| SET 4P | TCTCCCGAATCTCTCCTGTC | ATGTAGGGATTTCATGCCCA | 507 | 34022-34509 | 4 |
| SET 5P | CTGCTCCAGCCCAGTCAT | TTTTCTTTATCCCCACTATCAACA | 508 | 34481-34965 | 4 |
| SET 4D1 | TCAAGGACCACCACCACCCT | CCCAGCCTGAGACCCACATA | 726 | 34824-34965 | 4 |
| SET 5 | ATGCCTGAATTTTTCCCAAG | ACCTGACCCACATCTTCTGG | 781 | 37716-38496 | 5 |
| SET 6 | ACAGGGAGTGGGCTAAATAACAAAC | CACCCGGCCCAAGTTCATTC | 899 | 41216-42114 | 6,7 |
| SET 7A | AAGTTGCTTGGAGAGGATGGGA | TCTTCATCCGACTGCTGCTCTG | 627 | 55850-56476 | 8 |
| SET 9P | CCTAAAATCACACACCCAACAG | ACTGATTTAAACGGCTGGAA | 501 | 56217-56698 | 8 |
| SET 10P | TTCCTAAATTATGGGAAATCCTTTT | TGCATTATTTTTCCTTCTGACAAA | 462 | 56659-57103 | 8 |
| SET 7D | AGCCATCGTGTTGGTGAGTGT | GCCAGATAGCCAGATTTTCCTTTGC | 480 | 57008-57487 | 8 |
| Exp. Primer | GCCTGGTGTTAAGGGGAGAG | CAAGTCTTGAAGCATTTGTGGA | 660 | 216-875 | 2 |

**Supplemental Table 2. RFLP utilized to confirm the PCR amplicons prior to sequencing**

| **Exon number** | **Enzyme** | **Expected sizes** | |
| --- | --- | --- | --- |
| 1 | TAQαI | 121 bp | 452 bp |
| 3 | EcoR I | 255 bp | 377 bp |
| 5 | EcoRV | 339 bp | 442 bp |
| 6 and 7 *(in a single PCR amplicon product)* | EcoR I | 87 bp | 812 bp |
|  | Dpn I | 353 bp | 546 bp |
